# Glucose variability: a new risk factor for cardiovascular disease

**DOI:** 10.1007/s00592-023-02097-w

**Published:** 2023-06-21

**Authors:** Martina Belli, Alfonso Bellia, Domenico Sergi, Lucy Barone, Davide Lauro, Francesco Barillà

**Affiliations:** 1 Division of Cardiology, Department of Systems Medicine, Tor Vergata University, 00133 Rome, Italy; 2 Cardiovascular Imaging Unit, San Raffaele Scientific Institute, 20132 Milan, Italy; 3 Department of Systems Medicine, Tor Vergata University, 00133 Rome, Italy

**Keywords:** Diabetes, Hyperglycemia, Glucose variability, Atherosclerosis, Cardiovascular diseases

## Abstract

**Aims and data synthesis:**

Glucose variability (GV) is increasingly considered an additional index of glycemic control. Growing evidence indicates that GV is associated with diabetic vascular complications, thus being a relevant point to address in diabetes management. GV can be measured using various parameters, but to date, a gold standard has not been identified. This underscores the need for further studies in this field also to identify the optimal treatment.

**Conclusions:**

We reviewed the definition of GV, the pathogenetic mechanisms of atherosclerosis, and its relationship with diabetic complications.

## Introduction

Cardiovascular and cerebrovascular diseases are the leading cause of death worldwide [1]. Diabetes mellitus—alongside hypertension, hypercholesterolemia and smoking—is among the most relevant independent risk factors for coronary artery disease. The progression of atherosclerosis occurs earlier and more rapidly in subjects with hyperglycemia than in the general population; in accordance, people with type 2 diabetes mellitus (T2D) are twice as likely to have heart disease or a stroke than people without diabetes [2, 3]. Beyond macrovascular atherosclerotic complications, microvascular diseases affecting small vessels (namely diabetic retinopathy, nephropathy and neuropathy) play also a pivotal role in increasing the overall morbidity and mortality attributable to diabetes. Early recognition of diabetes itself and beginning of adequate treatment are therefore critical to mitigate the burden of disease determined by these micro and macrovascular complications [4, 5].

High blood glucose levels, especially when accompanied by augmented glucose fluctuations over the day, can affect various kinds of molecular mechanisms in various target cells and tissues [6], resulting in the leading cause of both micro- and macrovascular complications in the diabetic patient. At the macrovascular level, hyperglycemia induces a pro-inflammatory and pro-thrombotic state, promotes protein glycation and the formation of reactive oxygen species; all these are contributing factors for atherosclerosis. Additionally, diabetic microangiopathy itself can accelerate atherosclerosis. The vasa vasorum, small vessels located in the adventitia, respond to hyperglycemia-induced hypoxia and ischemia with neoangiogenesis that would appear to link the micro and macrovascular disease [7]. However, optimal glucose control has demonstrated clear beneficial effects on microvascular complications, but less on macrovascular ones, since multiple risk factors beyond glycemic control need to be addressed to substantially reduce cardiovascular events in diabetic patients [8, 9].

There is some evidence that cardiovascular atherosclerotic complications can occur in patients with impaired glucose homeostasis, even years before the onset of diabetes itself. For instance, in the "Glucose Tolerance in Acute Myocardial Infarction" prospective study, the presence of impaired glucose tolerance (i.e., the condition that occurs before the development of overt diabetes) was the main predictor of acute myocardial infarction and cardiovascular death [10]. More recently, increased blood glucose excursions over the day, including hypoglycemic periods and post-prandial increases, have gained attention in the pathogenesis of diabetic vascular complications, since this sort of glucose dysregulation seems to be significantly associated with vessel damage, perhaps more than mean blood glucose values themselves.

## Glucose variability

Glycated hemoglobin (HbA1c) is currently the gold standard biochemical parameter for the assessment of glycemic control and treatment efficacy in diabetic patients [11]. However, HbA1c is a measure of mean blood glucose concentrations [12, 13], not necessarily reflecting the short-term glycemic peaks and nadirs (lasting minutes or hours), especially in the post-prandial state, which could add or modify the risk of vascular damage [14]. As a matter of fact, two distinct diabetic patients with similar Hb1Ac values may have different glycemic fluctuations, and the individual risk of vascular complications may be highly affected by the extent of both post-prandial glycemic excursions and episodes of hypoglycemia [14]. For these reasons, the role of glucose variability (GV) has gained attention in recent years, in terms of significant association with cardiovascular disease risk in patients with T2D. A number of studies suggest that poor post-prandial blood glucose control may contribute to vascular risk, especially in those with established atherosclerotic disease, rather than chronic hyperglycemia per se [15–17]. Importantly, even though a certain degree of variability can be observed in subjects with normal glucose tolerance and without evidence of atherosclerotic disease, the impairment in post-prandial glucose excursions and consequent relationship with hemostatic and endothelial abnormalities is typical of individual with diabetes or impaired glucose regulation [16, 18]. For all these reasons, it is essential to establish the level above which GV is intended of pathological significance in order to recognize this abnormality as soon as possible and improve prevention of diabetic vascular complications. However, there is currently no consensus on how best assessing GV and defining when it is of pathological relevance, in order to help physicians improving the cardiovascular risk assessment in patients with T2D. Therefore, in this review, we examined the concept and definition of GV, the mechanisms by which it affects the development and progression of atherosclerosis, and its potential role as a predictor of CVD.

## Glycemic variability indicators

Previous definition of GV was rather generic, potentially including inter-day variability in fasting blood glucose, glycemic post-prandial peaks or glycated hemoglobin variability. More recently, this concept has been specified in more details, so that the widely accepted definition of GV considers the intra-day glycemic excursions, including episodes of hyperglycemia and hypoglycemia. It is characterized by the amplitude, frequency and duration of glucose deviations from the steady state during a certain period of time as measured by Continuous Glucose Monitoring (CGM) systems [19, 20]. There are numerous metrics to assess GV, some of them are simple to estimate and use in clinical practice, and others are more complex and less immediate [21]. However, no gold standard measure to fully evaluate GV has been identified to date. Basically, there are predominantly two types of GV according to the length of time-interval: (1)* long-term GV*, based on serial determinations over a longer period of time, involving serial fasting plasma glucose (FPG), post-prandial glucose (PPG) measurements and, less frequently, HbA1c values; (2)* short-term GV*, represented by both intra-day and inter-day GV [19–22], which is more closely related to the concept of glucose fluctuations, the risk of hypoglycemia and the adverse clinical outcomes of GV related to vascular damage [23, 24]. Some of the key metrics to express the amplitude of short-term GV are summarized in Table 1. The most immediate indexes to assess GV are calculated referring to the *glycemic mean values* over the day, in order to primarily capture mealtime-related glucose excursions [21]. *The standard deviation *(*SD*)*,* including total SD, intra-day SD and inter-day SD, is the subsequent index of dispersion of the data around the mean glycemic value and was initially found to be the simplest approach to assess glycemic variability. However, its use implies that glucose measures are normally distributed, which is typically not the case. Another measure designed to capture mealtime-related glucose excursions is the *mean amplitude of glucose excursion *(*MAGE*). This metric includes the mean of glycemic excursions from nadir to peak blood glucose levels and vice versa that are > 1.0 SD of blood glucose mean value [25]. Therefore, takes into account, glycemic peaks and nadirs occurring daily but does not account for the total number of fluctuations. It strongly depends on the sampling frequency, with no clear distinction between beginning and ending of peaks and nadirs**.** Further, it is questionable whether only mealtime excursions or excursions larger than 1.0 SD would have clinical importance. Other GV indices include the *J-index*, which is calculated from the mean blood glucose and SD [21]; the *low blood glucose index *(*LBGI*) and *high blood glucose index *(*HBGI*), which is designed to be sensitive to the frequency and severity of hypoglycemia or hyperglycemia; and the *average daily risk*, which is designed to predict both severe hyperglycemia and hypoglycemia [21]. However, all these methods are not adjusted for the mean blood glucose, whereas GV is known to be significantly influenced by the mean blood glucose over the considered period. For this reason, while most physicians being familiar with the use of SD in clinical practice, the preferred amplitude measure to assess GV in research is the *coefficient of variability (CoV)*, which is calculated from the SD divided by the mean glycemic value. CoV incorporates the advantage over SD of being a measure that takes into account mean blood glucose, thus being more descriptive of hypoglycemic excursions. Accordingly, some recent consensus statements for the interpretation of CGM data in diabetic patients recommends that CV should be used as the primary measure for assessing GV, with increased GV defined as CoV ≥ 36% [26, 27].

**Table 1:** Key metrics commonly used to express glucose variability

Amplitude of GV (temporal resolution range: hours to days)	Description
MAGE	Average of absolute differences between glucose peaks and nadirs (each difference needs to be greater than 1 SD from the mean). MAGE reflects within-day GV
SD	Variation around the mean blood glucose (intra-day or inter-day)
CoV = SD/mean	Magnitude of variability relative to mean blood glucose
LBGI	Measure of frequency and magnitude of hypoglycemia (amplifies hypoglycemic excursions without accounting for hyperglycemia)
HBGI	Measure of frequency and magnitude of hyperglycemia (amplifies hyperglycemic excursions without accounting for hypoglycemia)

Timing of GV based on CGM (temporal resolution range: minutes to hours)	Description
Time within, above or below target range	Quantitative measure of time spent within an individual’s target glucose range; time spent below this range; time spent above this range

*GV* Glucose variability; *MAGE* Mean amplitude of glucose excursion; *SD* Standard deviation; *CoV* Coefficient of variation; *LBGI* Low blood glucose index; *HBGI* High blood glucose index; *CGM* Continuous glucose monitoring

Finally, several metrics have been developed in order to additionally assess the so-called inter-day glycemic variability. Among them, the mean of daily differences (MODD)—namely the absolute difference in blood glucose levels at the same time on consecutive days—is widely used while having the limitation of being easily affected by the content and time of the different meals between the considered days [21]. The Ambulatory Glucose Profile (AGP) is used in daily practice using CGM. In the AGP, a curve showing the median blood glucose levels and a curve showing the 25 and 75 percentiles of blood glucose levels within a specified period, called the interquartile range (IQR), are drawn. The median, IQR and other values obtained using the AGP can be used to evaluate within-day and between-day glycemic fluctuations [28]. CGM technology can further expands the ability to assess glycemic control throughout the day, quantifying the time below, within, and above the established glycemic targets. The most important of these clinical metrics are the time in targeted blood glucose range (TIR), namely the percentage of time that a person spends with their blood glucose levels in a target range. The range varies depending on the person, but general guidelines suggest starting with a range of 70 to 180 mg/dl [29].

## Pathogenesis of cardiovascular complications in diabetes

**Fig. 1:**
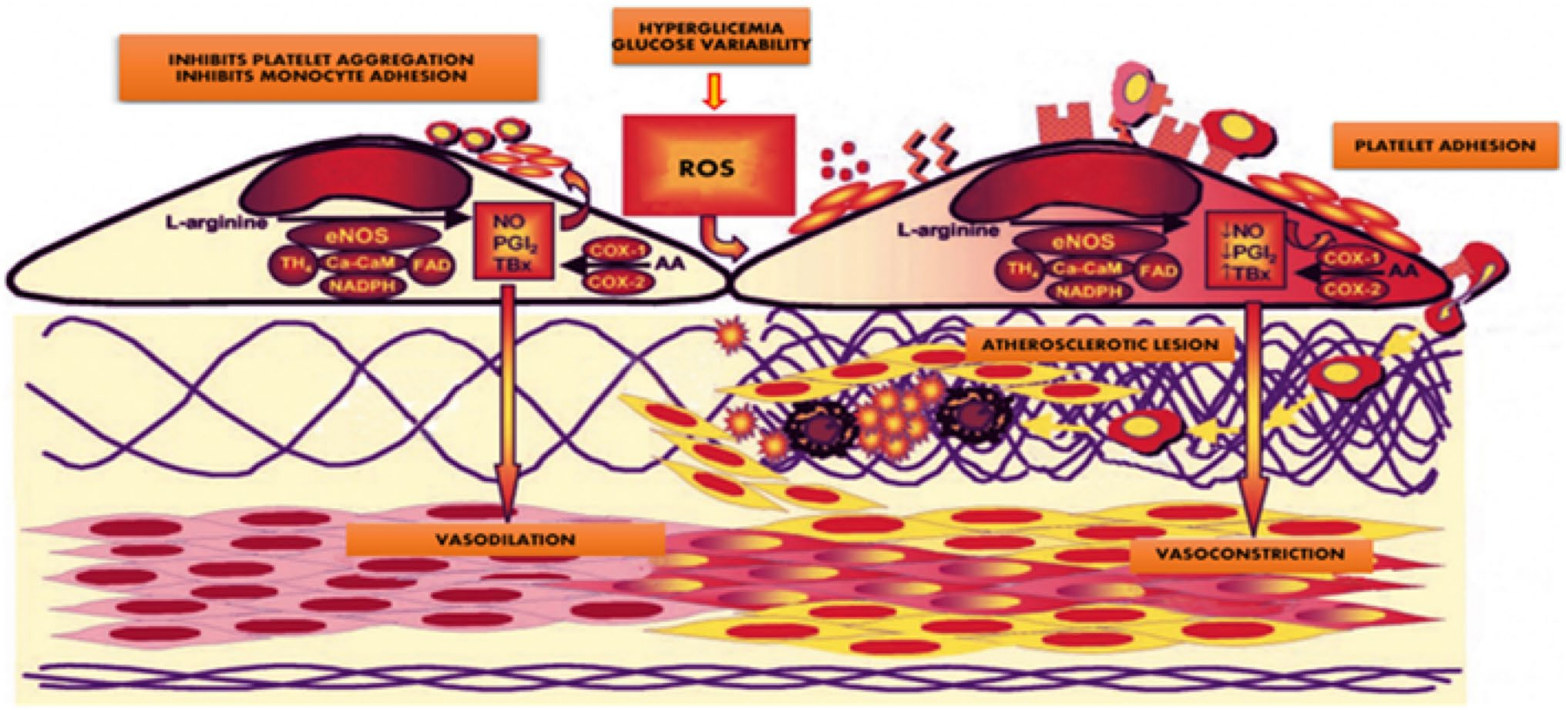
Hyperglycemia and glucose variability directly impair endothelial function by increasing local reactive oxygen species production. They accelerate nitric oxide decay, damping its vasodilator effect. Endothelial dysfunction causes expression of adhesion molecules. Platelets and monocytes migrate into intima. Monocytes first turn into macrophages and then into foam cells, contributing to the formation of atherosclerotic plaque. Platelets and macrophages activates secrete growth factor like PDGF, EGF, TGF α that lead smooth muscle cells proliferation. They synthesize collagen, elastin forming extracellular matrix. All this plays a key role in the development of atherosclerosis. *ROS* Reactive Oxygen Species; *NADPH* Nicotinamide Adenine Dinucleotide Phosphate; *NO* Nitric Oxide; *FAD* Flavin Adenine Dinucleotide; *eNOS* Endothelial Nitric Oxide Synthase; *PGI2* Prostaglandin I2; *TBX* Thromboxane; *COX1-2* Cyclooxygenase; *NF-κB* Nuclear Factor kappa-light-chain-enhancer of activated B cells

Chronic hyperglycemia and GV are both important markers of endothelial and cardiovascular damage even in patients with diabetes of short duration and optimal glycemic control. In fact, they increase the production of reactive oxygen species (ROS), which inactivate nitric oxide (NO), leading to endothelial dysfunction and vascular complications. ROS interact with protein, lipid and DNA generating numerous oxidative products [30]. Among these, nitrotyrosine and 8-hydroxydeoxyguanosine (8-OHdG) are responsible for the extent of vascular damage induced by periodic or continuous exposure to highly variable glycemic levels [31]. The assessment of events downstream of ROS generation, such as cell apoptosis, nuclear factor (NF) kB activation in mononuclear cells, cell growth and collagen synthesis, in cultured human renal tubulointerstitial cells [32, 33], has helped demonstrate endothelial pathological effects sustained by the variability of blood glucose concentration. Quagliaro et al. examined the different effect that GV and consistently high level of blood glucose exert on the generation of ROS, by measurement of nitrotyrosine and 8-OHdG, and the consequent effects of oxidative stress on cell apoptosis. They showed that apoptosis is markedly increased in human umbilical vein endothelial cells (HUVECs) when exposed to periodic changes in glucose concentration, compared with those exposed to constant high concentrations [34]. Furthermore, GV increases protein kinase C (PKC) activity, enzyme whose main action is to transfer a phosphate group of ATP to a hydroxyl group on a serine, threonine or tyrosine of the target molecule. This phosphorylation is essential for the performance of a variety of intracellular protein functions. Hyperglycemia leads to an increase in diacylglycerol (DAG) content, which in turn activates various PKC isoforms, with transduction of several intracellular signals that alter gene expression of numerous pro-atherogenic factors. Activation of PKC increases the production of several cytokines, extracellular matrix, fibrinolytic-acting PAI-1(Plasminogen activator inhibitor-1) and ET-1(endothelin 1), as well as stimulating the production of vascular endothelial growth factor (VEGF) [35] and of vasoconstrictors (Fig. 1). Another important mechanism in determining the endothelial dysfunction is the oxidation of low density lipoproteins (LDLs) [36], which has been shown to associate with mean glucose excursions in prospective studies [37]. Increased oxidative stress facilitates LDLs oxidation mechanisms, with formation of so-called ox-LDLs, which are able to penetrate into the subendothelial layers where they activate monocytes that first turn into macrophages and then into foam cells, contributing to the formation of atherosclerotic plaque. Ox-LDLs cause activation of endothelial cells, have cytotoxic effects on the endothelium and stimulate the production of several cell growth factors and adhesion molecules. Ox-LDLs also cause activation of several pro-inflammatory genes, increase platelet aggregation and thrombogenesis [38]. Taken together, all these mechanisms significantly contribute to the development and progression of atherosclerosis in patients with T2D. Endothelial dysfunction, furthermore, in patients with insulin resistance and pre-diabetes causes coronary disease with consequent highest rate of major adverse cardiovascular events (MACEs) and worse prognosis despite optimal medical therapy and vessels revascularization [39]. Endothelial dysfunction, so, plays a key role in the pathogenesis of the diabetic vascular complications but only partially explains the increased risk of coronary artery disease (CAD) in diabetic patients. Recent findings suggest a potential involvement of epigenetics, including DNA methylation, histone modifications and noncoding RNA control (ncRNA); indeed, hyperglycemia can also induce epigenetic modifications that lead to increased endothelial dysfunction and atherosclerosis.

Hyperglycemia determines DNA demethylation in endothelial cells through an upregulation of TETs. Hyperglycemia activates the NFκB-p65 gene in endothelial cells by mono-methylation of lysine 4 on histone 3 (H3K4m1) through histone methyltransferase Set7 and demethylation of H3K9 on the p65 promoter by lysine-specific demethylase 1 [40]. The NFκB upregulation causes increase in adhesion molecules, cytokines and chemokines, resulting in inflammation and atherosclerosis. Hyperglycemia also induces TET-2 mediated DNA demethylation changes that are involved in the differentiation of the vessel smooth muscle cells in a phenotype characterized by loss of contractility and increased proliferation and secretion of extracellular matrix proteins. Hyperglycemia causes reduction in the GPx-1 gene, which encodes the glutathione peroxidase-1 enzyme, crucial in preventing oxidative stress and endothelial dysfunction. The knowledge of epigenetic mechanisms, so, it might help to predict the development and progression of diabetes complications [41].

## Clinical implications

There is ample evidence that glycemic control is crucial in preventing CVD and reducing mortality. Since the 1990s, with the UKPDS [42] (UK Prospective Diabetes Study Group) and DCCT [43] (The Diabetes Control and Complications Study) studies, it has been observed that adequate glycemic control significantly contributes to reduce the occurring of cardiovascular complications in diabetic patients. The results of the UKPDS trial, which enrolled more than 3000 diabetic patients without cardiovascular disease at baseline and followed-up for 10 years, demonstrated the importance of tight glycemic control (defined as fasting blood glucose < 100 mg/dl) in reducing the incidence of microvascular complications, and with a trend toward a slightly non-significant 16% relative risk reduction in myocardial infarction. Such differences in the risk reduction between micro- and macrovascular complications in the UKPDS trial have been attributed to various and heterogeneous pathophysiological mechanisms. This because hyperglycemia and GV are likely to play a major role in the pathogenesis of microvascular complications, whereas several other extra-glycemic factors—including hypertension, smoking, dyslipidemia and overweight—are perhaps more directly involved in the development of atherosclerosis. This hypothesis was confirmed by the results of the STENO-2 trial [44], which showed that intensified multifactorial intervention—with tight glucose regulation as well as the use of renin–angiotensin system blockers, aspirin and lipid-lowering agents—determined major beneficial effects with respect to cardiovascular events compared with conventional therapy. The role of tight glycemic control in reducing microvascular complications of diabetes was clearly demonstrated by the DCCT/EDIC study [45], which enrolled only patients with type 1 diabetes who are known to suffer of greater glycemic excursions and GV than those with T2D. After a median follow-up of 6.5 years, researchers observed that intensive insulin treatment, with the goal of maintaining blood glucose concentrations close to the normal range, reduced the progression of diabetic retinopathy, nephropathy and neuropathy compared with conventional therapy. When looking at a population of patients with longstanding T2D and high cardiovascular risk, as those enrolled in the ADVANCE (Action in Diabetes and Vascular Disease) trial were [46], a strategy of intensive glucose control involving gliclazide and other drugs as required, yielded a significant risk reduction in microvascular complications (primarily nephropathy) but no significant reduction in macrovascular complications. Of note, following analysis, of the dataset of the ADVANCE trial, showed that visit-to-visit variability of fasting glucose, as well as of HbA1c, were significantly related with vascular events and to an increased risk of mortality [47]. Similar results were reported by The Veterans Affairs Diabetes Trial (VADT) [48] and by the Action to Control Cardiovascular Risk in Diabetes (ACCORD) trial [49], which both failed to demonstrate in the primary analysis a clear beneficial effect of intensive vs standard anti-hyperglycemic therapy on cardiovascular events, at least in patients with long duration of diabetes. The ACCORDION study evaluated the long-term effects of the ACCORD therapeutic strategy and demonstrated an increase in cardiovascular mortality, a result already largely demonstrated in the ACCORD study, but did not show an increase in both mortality and non-fatal cardiovascular events. The mechanisms leading to increased mortality are still to be elucidated; however, it appears that the absence of diabetic retinopathy is an important predictor of the beneficial effect of intensive glucose control on the risk of cardiovascular disease and possibly death [50]. However, a secondary analysis of the VADT trial including coefficient of variation (CV) and average real variability (ARV) for fasting glucose and HbA1c, both measured every three months for up to 84 months, showed that variability of fasting glucose was significantly associated with CVD events, and this relationship was particularly evident in patients receiving intensive glucose control [51].

In the end, while these secondary analysis from RCTs being not fully conclusive in disentangle the contribution of CV toward the risk of coronary heart diseases, there is some experimental evidence supporting the role of CV in the pathogenesis of atherosclerosis. In accordance, the effect of GV on atherosclerotic plaque morphology has been experimentally investigated in patients with coronary artery disease using virtual histology intravascular ultrasound [52]. In this study, GV was expressed as the mean amplitude of glycemic excursions (MAGE index) and was significantly associated with the volume of necrotic core in the atherosclerotic plaque. Moreover, glucose fluctuation and hypoglycemia turned out to be the only independent predictor of the formation of thin cap fibroatheroma, which characterizes the vulnerable plaque at higher risk of rupture [52], even in patients with coronary artery diseases taking intensive lipid-lowering therapy [53]. D’Onofrio et al. analyzed hyperglycemic thrombi in STEMI patients showing that they have a higher size and increased miR33, reactive oxygen species and pro-inflammatory markers and a lower endothelial SIRT1 expression. The miR33/SIRT1, thus, pathway is responsible for the increased pro-inflammatory and pro-coagulable state of coronary thrombi in hyperglycemic STEMI patients [54]. Paolisso et al. analyzed the link between stress hyperglycemia, infarct size and inflammatory burden in diabetic patients with acute myocardial infarction (AMI) treated with Sodium-glucose Cotransporter 2 Inhibitors (SGLT2-I), compared with other oral antidiabetic (OAD) agents. SGLT2-I would also appear to play a key cardioprotective role in acute coronary syndromes (ACS), independent of glycemic control; in fact, patients treated with SGLT2-I showed significantly lower inflammatory burden and infarct size than those treated with OAD agents [55].

So, is tight glycemic control during ACS useful? Studies are scarce and sometimes controversial, particularly because of the risk of hypoglycemia due mainly to insulin therapy. SGLT2-I and Glucagon-Like Peptide-1 Receptor Agonists (GLP1-RA) would seem to be the solution to this problem, as they reduce the risk of hypoglycemia and have cardioprotective pleiotropic effects not only in the cardiovascular prevention, but also in acute conditions, such as ACS [56].

To date, these two drug classes also appear to be the only ones to improve coronary microvascular dysfunction (CMD), a condition that results in a significantly worse prognosis with a higher risk of MACES at 5 years [57]. Until a few years ago, CMD therapy consisted in controlling cardiovascular risk factors, but the results of preclinical studies on the effects of SGLT2-I and GLP1-RA in this field are decidedly encouraging. However, further studies are needed to confirm their role on microcirculation and to identify the precise mechanisms [58, 59].

The anti-inflammatory effect of SGLT2-I would be implicated in improving autonomic dysfunction in patients with vasovagal syncope (VVS). It could consequently result in a lower recurrence rate of VVS in SGLT2-I users than in non-SGLT2-I users at 1-year follow-up [60]. It had already been hypothesized that cardiac autonomic dysfunction could lead to a higher recurrence rate of VVS in patients with T2DM compared to non-diabetics; therefore, it is essential to understand the treatment to reduce the alterations of the autonomic function [61]. The negative effect of hyperglycemia on sympathetic tone not only worsens the prognosis in patients with syncope, but also in Takotsubo Syndrome (TTS). Hyperglycemia would cause a chronic alteration of cardiac metabolism with impaired norepinephrine reuptake and consequent more severe sympathetic denervation [62].

Taking together, this experimental evidence may support the role of GV and hyperglycemia in the development of diabetic cardiovascular complications, and its relevance as further target of glycemic control in order to reduce the burden of atherosclerotic vascular disease and autonomic dysfunction in patients with T2DM.

## Glucose variability and macrovascular complications: state of the art

In the last decade, techniques have been developed that allow CGM [63] mainly used by patient with T1DM and in 2014, the flash glucose monitoring (FGM) system was introduced [64].

Recently, it has been reported that high GV is associated with the development and progression of diabetic vascular complications. Several studies have shown that long-term GV, in particular HbA1c and fasting plasma glucose variability, was associated with increased risk of macrovascular events [65] such as myocardial infarction, stroke, peripheral artery disease and all-cause mortality. Short-term GV, especially CGM, was associated with carotid intima-media thickness [66], high arterial stiffness [67], all-cause mortality and CV mortality [68]. In addition, higher CGM and lower CGM time in range (TIR) were associated with higher carotid-femoral pulse wave velocity [67].

Some data suggest that diabetic management through CGM may also improve clinical outcomes and reduce the risk of complications. Accordingly, the American Diabetes Association and the Italian National Health Service (NHS) have recommended the use of CGM for the management of patients with T1DM and T2DM treated with multiple daily insulin injections [69].

Recently, a survey of diabetologists and cardiologists was conducted to obtain expert consensus on the use of CGM in diabetic patients at high risk of CV or with a history of CV events [70]. The experts agreed that CGM is a prognostic tool for T1DM and T2DM treated with multiple daily insulin injections. They strongly believe that TIR provides more information than HbA1c and that, this is a useful tool to optimize the treatment of people with heart disease.

While the clinical benefits of CGM are established, studies about FGM are scarce, in fact to date there is no evidence of its possible role in reducing macrovascular complications.

## Conclusion

In this review, we highlighted the role of glucose variability in the pathogenesis of atherosclerotic disease, describing potential mechanisms and summarizing the results of the main supporting studies currently available. While GV being a physiological phenomenon in the context of glucose homeostasis regulation, fluctuations of blood glucose can be extremely enhanced in patients with diabetes, thus contributing not only to the increased mean blood glucose values but also to the development of chronic vascular complications. Although there is no definitive demonstration from RCTs, a growing body of evidence suggests that GV contributes to the development of CVD through different pathogenetic mechanisms. Several metrics are currently available to express GV but, to date, the gold standard has not been identified, universal definition is lacking and the role of CGM and FGM on macrovascular complications is also uncertain. This underscores the need for further studies in this field, in order to better define the practical use of GV as new efficacy goal for diabetic treatment, over and above standard parameters of glycemic control.

## Data Availability

No new data were created or analyzed in this study. Data sharing is not applicable to this article.

## Abbreviations

GV: Glucose variability
HbA1c: Glycated hemoglobin
ACS: Acute coronary syndrome
CVD: Cardiovascular disease
T2D: Type 2 diabetes
SMBG: Self-monitoring of blood glucose
CGM: Continuous glucose monitoring
FPG: Fasting plasma glucose
MAGE: Mean amplitude of glucose excursion
MODD: Mean of daily differences
AGP: Ambulatory glucose profile
TIR: Time in range
UKPDS: UK prospective diabetes study group
DCCT: The diabetes control and complications study
ADVANCEE: Action in diabetes and vascular disease
VADT: Veterans affairs diabetes trial
